# Smoking and Multiple Sclerosis: An Updated Meta-Analysis

**DOI:** 10.1371/journal.pone.0016149

**Published:** 2011-01-13

**Authors:** Adam E. Handel, Alexander J. Williamson, Giulio Disanto, Ruth Dobson, Gavin Giovannoni, Sreeram V. Ramagopalan

**Affiliations:** 1 Wellcome Trust Centre for Human Genetics, University of Oxford, Oxford, United Kingdom; 2 Department of Clinical Neurology, University of Oxford, Oxford, United Kingdom; 3 Department of Physical and Theoretical Chemistry, University of Oxford, Oxford, United Kingdom; 4 Barts and The London School of Medicine and Dentistry, Blizard Institute of Cell and Molecular Science, Queen Mary University of London, London, United Kingdom; National Institutes of Health, United States of America

## Abstract

**Background:**

Multiple sclerosis (MS) is a leading cause of disability in young adults. Susceptibility to MS is determined by environmental exposure on the background of genetic risk factors. A previous meta-analysis suggested that smoking was an important risk factor for MS but many other studies have been published since then.

**Methods/Principal Findings:**

We performed a Medline search to identify articles published that investigated MS risk following cigarette smoking. A total of 14 articles were included in this study. This represented data on 3,052 cases and 457,619 controls. We analysed these studies in both a conservative (limiting our analysis to only those where smoking behaviour was described prior to disease onset) and non-conservative manner. Our results show that smoking is associated with MS susceptibility (conservative: risk ratio (RR) 1.48, 95% confidence interval (CI) 1.35–1.63, p<10^−15^; non-conservative: RR 1.52, 95% CI 1.39–1.66, p<10^−19^). We also analysed 4 studies reporting risk of secondary progression in MS and found that this fell just short of statistical significance with considerable heterogeneity (RR 1.88, 95% CI 0.98–3.61, p = 0.06).

**Discussion:**

Our results demonstrate that cigarette smoking is important in determining MS susceptibility but the effect on the progression of disease is less certain. Further work is needed to understand the mechanism behind this association and how smoking integrates with other established risk factors.

## Introduction

Multiple sclerosis (MS) is a complex neurological condition characterised by demyelination and axonal loss.[1] The nature *versus* nurture argument has largely settled into a model in which many genetic and environmental factors interact at different times prior to and following the clinical onset of MS to determine susceptibility and the clinical phenotype expressed.[2] These factors are currently thought to include the Human Leukocyte Antigen (HLA) region, smoking, Epstein-Barr virus (EBV) and vitamin D.[3], [4], [5]

Early case-control studies suggested that a history of smoking could play a role in MS susceptibility, but either fell slightly short of formal significance or analysed a large number of possible variables simultaneously.[6], [7] Since then a number of case-control and population cohort studies have been performed with near universal agreement that smoking cigarettes increases susceptibility to MS.[4], [8], [9], [10], [11], [12], [13], [14], [15], [16], [17], [18] Evidence from New Zealand suggests that smoking behaviour may contribute to the latitudinal distribution of MS risk there.[19], [20]

A previous meta-analysis of six studies published in 2007 demonstrated an overall risk ratio (RR) of 1.24 (95% confidence interval (CI) 1.04–1.48) with a p-value of 0.01 in the most conservative model used.[21] Since this was published other much larger studies have been directed towards understanding the association between smoking cigarettes and MS susceptibility. There is also evidence from analysing several case-control cohorts in parallel that smoking behaviour does not change significantly following diagnosis, suggesting that studies examining current smoking behaviour may also be informative.[4] Risk prediction in MS is a nascent field but accurate estimates of disease susceptibility in relation to environmental factors are clearly important for the success of any such predictions.[22] The relationship between smoking and secondary progression is currently highly controversial, with some studies suggesting an increased risk of progression and others showing no effect.[12], [23], [24], [25], [26]

In this study we aimed to broaden the previous meta-analysis to include all subsequently published and informative studies of smoking behaviour and MS risk. Secondarily we aimed to establish any correlation between both smoking and latitude and between smoking and the risk of secondary progression in MS.

## Methods

### Search Strategy

We searched OLDMEDLINE and MEDLINE from 1960 to May 2010 with the phrase: “(smok* OR cigarett*) AND (multiple sclerosis OR ms)”. We hand-searched abstracts generated from this search term for cohort or case-control studies and examined references of these articles for potential additional studies. We included studies if they reported smoking behaviour, were either case-control or cohort studies, and had a dedicated control group. Studies were excluded if they gave insufficient data to calculate 95% CIs for RRs or smoking behaviour varied throughout the reported study period. Experts in the field were unaware of any ongoing unpublished studies for inclusion.

### Statistical analysis

We used the generic inverse variance with random effects model in Reference Manager 5.0 to calculate the overall RR, 95% CI and test statistic for the interaction and heterogeneity of studies. Using the rare disease assumption, we combined estimates of odds ratio (OR) and RR [27]. For papers not reporting RR and 95% CIs but with sufficient information to calculate these, we used the methods described in [28]. We performed meta-analysis separately using a conservative (including only studies where smoking behaviour was described prior to disease onset) and non-conservative (all studies regardless of whether smoking behaviour was prior to onset or current) models. RRs were subsequently calculated for subgroups of studies and compared between case-control and cohort studies. We extracted latitude from papers based on either the latitude of the study centre if performed in a distinct region or the geographical midpoint of the country if a national study. We tested for an interaction between latitude and sex-ratio by both a comparison of RRs based on the median value for each factor and also performed linear weighted fit on the natural log of RRs in MATLAB.

## Results

### Included studies in smoking and MS susceptibility

Our search criteria produced 1,490 studies. We identified 14 studies for inclusion in the MS susceptibility arm of the meta-analysis.[4], [6], [7], [8], [9], [10], [11], [12], [13], [14], [15], [16], [17], [18] The details of the studies included are shown in **supplementary table S1**. These studies represent data from 3,052 cases and 457,619 controls. We excluded one study reporting smoking throughout pregnancy as the frequency of smoking changed throughout the study period.[29] We excluded two studies because insufficient data was described to calculate 95% CI for RR and the authors could not be contacted.[30], [31] Two additional studies were excluded because they did not include a control group.[32], [33] A number of studies were not included as they contained duplicate smoking data on a cohort already included in the meta-analysis.[34], [35], [36], [37] We also excluded the duplicate data published in the paper by Simon *et al*.[4]

### Smoking and MS susceptibility

**Figure 1:**
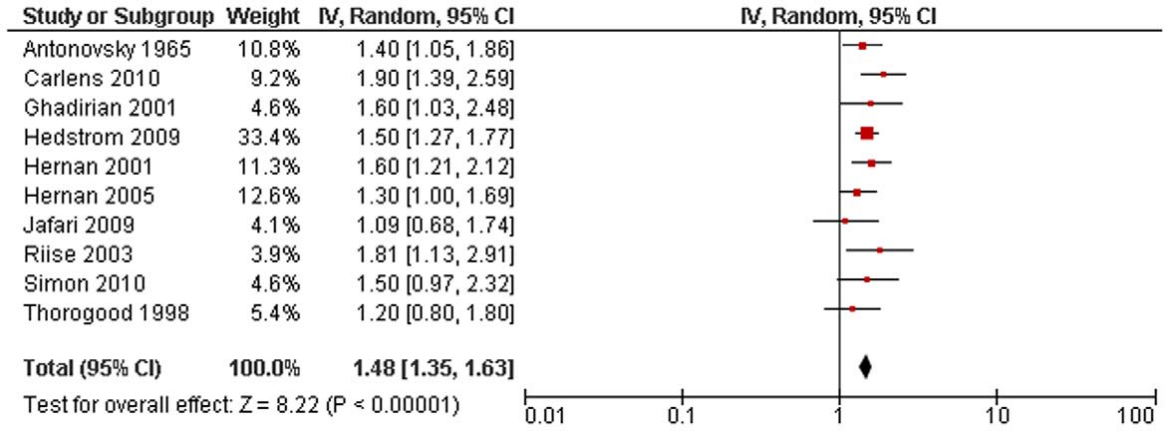
Forest plot of smoking and multiple sclerosis risk (conservative model).

**Figure 2:**
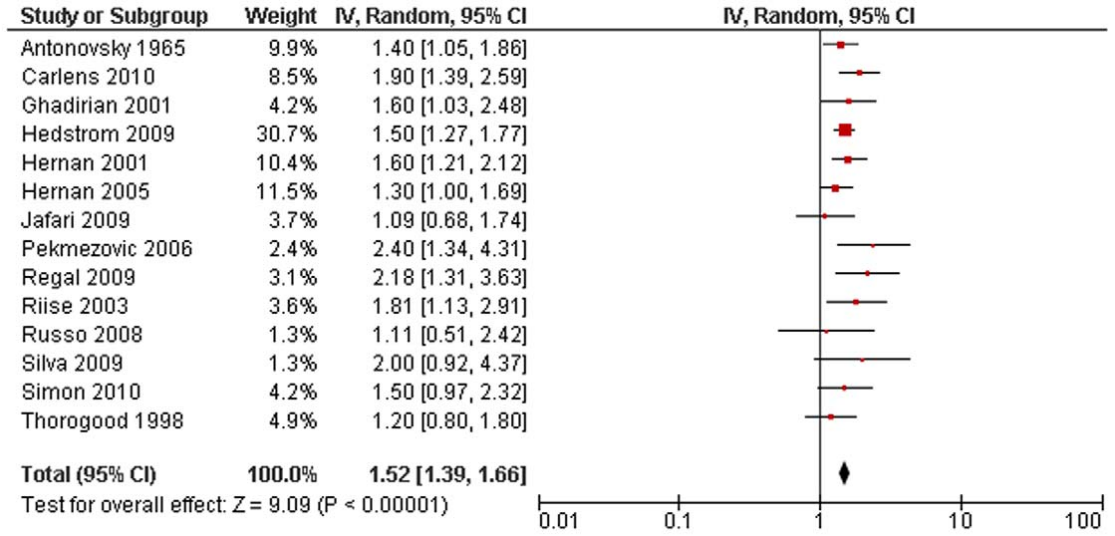
Forest plot of smoking and multiple sclerosis risk (non-conservative model).

**Figure 3:**
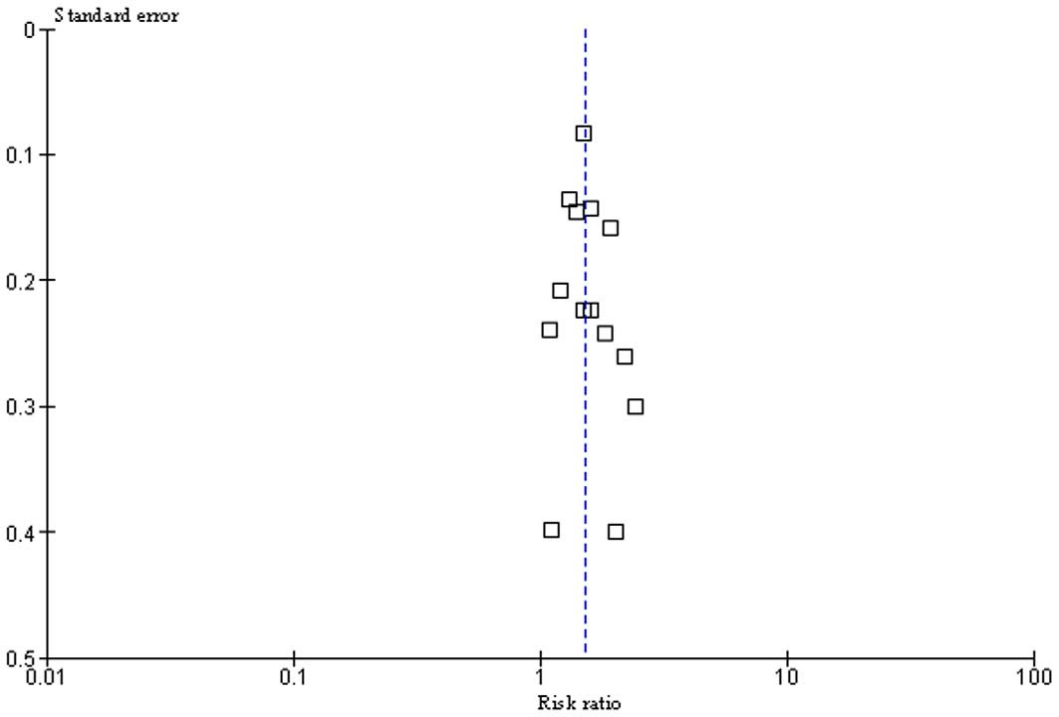
Funnel plot of all 14 susceptibility studies. Overall (non-conservative) meta-analysis risk ratio is shown as a dotted line.

10 studies were suitable for inclusion in the conservative model of smoking and MS risk. This showed a highly significant increased risk of MS associated with smoking with no significant statistical heterogeneity (RR 1.48, 95% CI 1.35–1.63, p<10^−15^, heterogeneity: χ^2^ = 7.38, p = 0.60; **figure 1**). Including all 14 studies in the non-conservative model yielded very similar results (RR 1.52, 95% CI 1.39–1.66, p<10^−19^, heterogeneity: χ^2^ = 12.99, p = 0.45; **figure 2**). There were no significant differences detected when comparing subgroups of studies based on design or diagnostic criteria, or when dichotomised based on median latitude or sex-ratio (excluding studies looking exclusively at either males or females) (**table 1**). Effect sizes of individual studies appeared to be uniformly distributed around the meta-analysis effect size, suggesting no large degree of publication bias (**figure 3**).

**Table 1:** Relative risk and 95% confidence intervals for different subgroups of studies.

Subgroup of studies	Relative risk	Lower 95% CI	Upper 95% CI	number of studies	p-value for comparison
Case-control	1.49	1.35	1.66	11	0.65
Cohort	1.58	1.25	2.00	3	
Interview/medical records smoking data	1.48	1.24	1.76	6	0.71
Questionnaire/unclear smoking data	1.54	1.38	1.72	8	
Diagnostic criteria	1.50	1.31	1.70	8	0.66
Physician/self-reported/methods not reported	1.57	1.34	1.84	6	
Latitude <46	1.61	1.37	1.88	7	0.41
Latitude ≥46	1.48	1.30	1.67	7	
Sex-ratio <2.13	1.46	1.14	1.87	5	0.89
Sex-ratio ≥2.13	1.49	1.32	1.69	5	

P-values for comparison between subgroups are also shown.

### Interaction with latitude and sex-ratio

**Figure 4:**
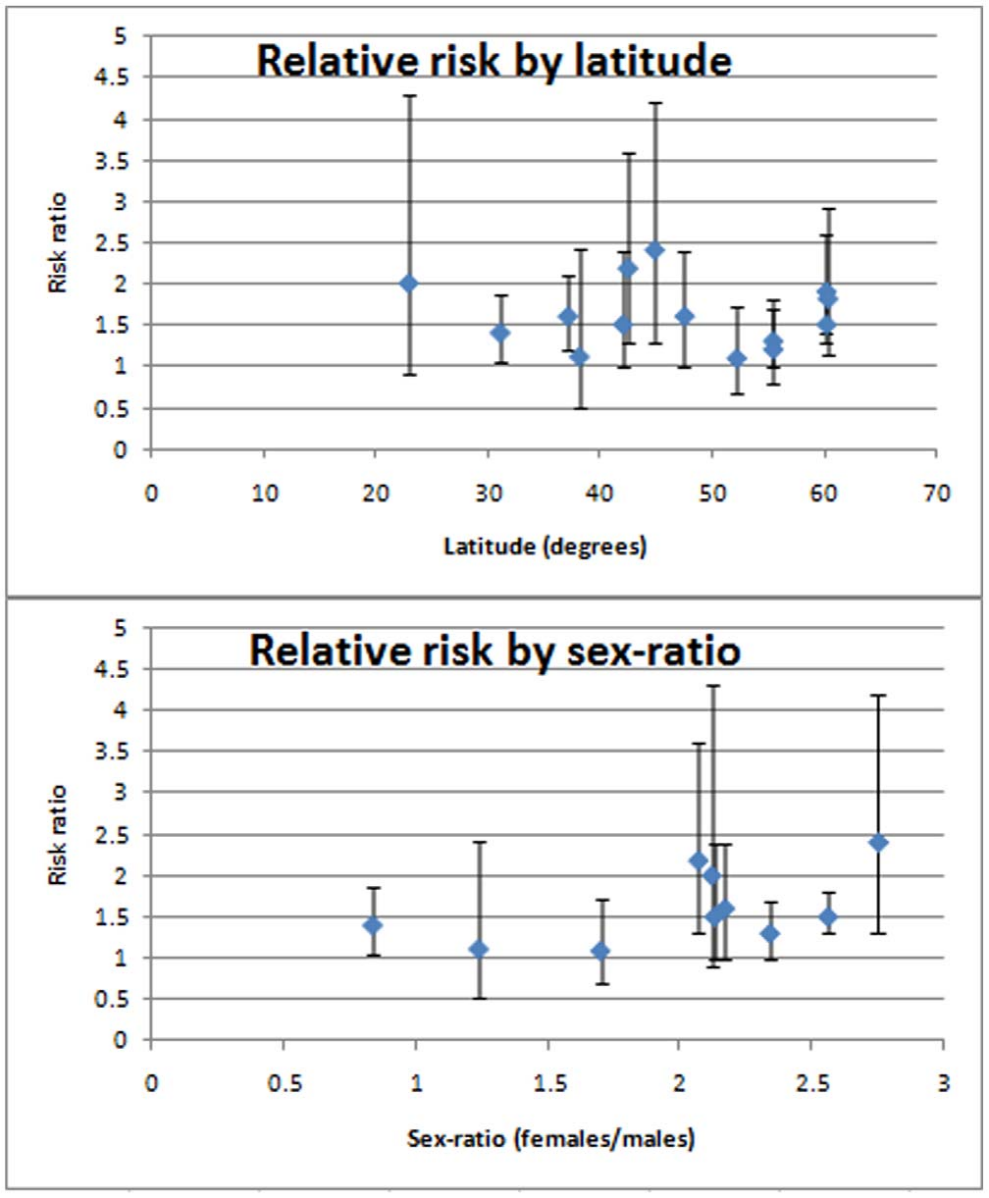
Plots of relative risk against latitude and sex-ratio. Error bars show 95% confidence intervals of relative risk estimates.

We tested both latitude and sex-ratio for correlation with the natural log of RR for each study using linear weighted fit (**figure 4**). No significant correlation was found for either (latitude: r^2^<0.01, p = 0.99; sex-ratio: r^2^ = 0.09, p = 0.40).

### Smoking and secondary progression in MS

**Figure 5:**
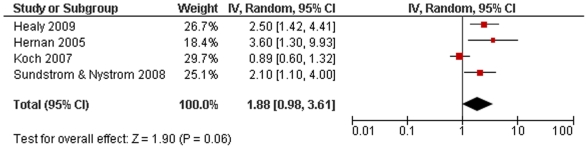
Forest plot of smoking and risk of secondary progressive multiple sclerosis.

We located 5 studies reporting data on secondary progression in MS and smoking behaviour.[12], [23], [24], [25], [26] Pittas and colleagues gave RR per pack-year rather than for smokers *versus* non-smokers and so this could not be included in the final analysis.[26] Our meta-analysis of the remaining 4 studies (**supplementary table S1**) showed a smoking RR for progression from relapsing-remitting MS to secondary progressive MS that fell just short of significance as well as highly significant statistical heterogeneity (RR 1.88, 95% CI 0.98–3.61, p = 0.06, heterogeneity: χ^2^ = 13.76, p = 0.003; **figure 5**).

## Discussion

Our meta-analysis includes data on a large number of MS cases and controls. It is firmly established that smoking cigarettes is associated with MS susceptibility but evidence suggests that this environmental factor alone is unlikely to account for either the global variation in prevalence.

The quality of studies varies widely as detailed in **table S1**. Our sensitivity analysis suggests that the specific diagnostic criteria or type of smoking data used make little difference to the strength of the relationship between MS susceptibility and smoking (**table 1**). Although most studies controlled for many confounding variables such as age, sex and latitude, it must be acknowledged that other, unknown confounders could be responsible for the effect observed. We find no effect of latitude on risk of MS with smoking; however differing degrees of migration in individual study populations may mean that we have missed a latitudinal effect.

A key aspect in establishing causation of any environmental factor is an interaction between susceptibility and dose [38]. Many of the studies detailed in this meta-analysis did demonstrate increasing MS susceptibility with increasing numbers of cigarettes smoked [6], [9], [10], [11], [16] but this was not a universal finding [7], [13]. The significant association between the degree of susceptibility to MS and pack-years smoked suggests that smoking may have more of an effect on susceptibility earlier in life, although without the exact numbers of cigarettes per day available, this is difficult to assess [11]. Unfortunately heterogeneity in how this data was recorded (pack-years, cigarettes per day etc.) renders meta-analysis of these aspects of studies problematic.

Our results are suggestive of an association between smoking and risk of secondary progressive MS, but the high level of heterogeneity between studies, both in design and statistical outcomes, limits the ability to draw firm conclusions. However, the demonstration of a dose-response effect on disease progression and disease severity is suggestive of a causal link [25], [26]. Several studies demonstrate that smoking at an early age increases the probability of progressive and severe MS [25], [26].

The mechanism by which smoking affects the susceptibility to and progression of MS is unclear. From population-based studies of tobacco snuff use, which does not appear to increase the risk of MS, it does not appear to be mediated solely by nicotine.[8], [10] It seems more likely that the mechanism involves components of cigarette smoke, a hypothesis supported by a study showing that the risk of MS may be increased with biochemical evidence of passive smoke exposure.[39] One highly attractive molecular candidate is nitric oxide, which has putative roles in demyelination and axonal loss.[40], [41] Cigarette smoke has a well-established role in susceptibility to autoimmune diseases, making a biological effect on the immune system highly probable.[42] Animal models have suggested that smoke exposure affects several compartments of the immune system, including innate immunity, B- and T-lymphocytes, and natural killer cells.[43], [44], [45] In rheumatoid arthritis, an autoimmune disease in which smoking is important in determining susceptibility, an interaction was identified between *HLA* alleles, cigarette smoking and immunity to α-enolase.[46] It is possible that the mechanism linking smoking and MS susceptibility may involve similar autoimmunity against proteins with post-translational modifications. Another possibility is that smoking could increase the risk of upper respiratory tract infections, an established risk factor for MS [47], [48].

As well as elucidating the mechanism(s) through which smoking affects MS susceptibility and, potentially, progression, future studies should investigate how environmental factors interact to determine MS risk. Simon and colleagues have already shown that high titres of antibodies against EBV antigens interact in a multiplicative manner with smoking on MS susceptibility, although the mechanism by which this occurs remains unproven.[4] Although Simon *et al.* did not find an interaction between *HLA* alleles and smoking, evidence exists in other autoimmune diseases that this may occur.[46], [49], [50] Also of interest would be the any alteration in sex specific aspects of MS risk following smoking and whether this risk has changed over time. Given the increasing sex-ratio observed in MS, it is possible that altered smoking behaviour may explain at least part of this trend [51], [52]. It is only by combining risk factors that patient-centred prediction of MS susceptibility will become a possibility.

## Supporting Information

Table S1 **Characteristics of studies included in the meta-analysis.**
(DOC)

## References

[1] Noseworthy JH, Lucchinetti C, Rodriguez M, Weinshenker BG (2000). Multiple sclerosis.. N Engl J Med.

[2] Handel AE, Giovannoni G, Ebers GC, Ramagopalan SV (2010). Environmental factors and their timing in adult-onset multiple sclerosis.. Nat Rev Neurol.

[3] Handel AE, Handunnetthi L, Giovannoni G, Ebers GC, Ramagopalan SV (2010). Genetic and environmental factors and the distribution of multiple sclerosis in Europe.. Eur J Neurol.

[4] Simon KC, van der Mei IA, Munger KL, Ponsonby A, Dickinson J (2010). Combined effects of smoking, anti-EBNA antibodies, and HLA-DRB1*1501 on multiple sclerosis risk.. Neurology.

[5] Ramagopalan SV, Maugeri NJ, Handunnetthi L, Lincoln MR, Orton SM (2009). Expression of the multiple sclerosis-associated MHC class II Allele HLA-DRB1*1501 is regulated by vitamin D.. PLoS Genet.

[6] Thorogood M, Hannaford PC (1998). The influence of oral contraceptives on the risk of multiple sclerosis.. Br J Obstet Gynaecol.

[7] Antonovsky A, Leibowitz U, Smith HA, Medalie JM, Balogh M (1965). Epidemiologic Study of Multiple Sclerosis in Israel. I. An Overall Review of Methods and Findings.. Arch Neurol.

[8] Carlens C, Hergens MP, Grunewald J, Ekbom A, Eklund A (2010). Smoking, use of moist snuff, and risk of chronic inflammatory diseases.. Am J Respir Crit Care Med.

[9] Ghadirian P, Dadgostar B, Azani R, Maisonneuve P (2001). A case-control study of the association between socio-demographic, lifestyle and medical history factors and multiple sclerosis.. Can J Public Health.

[10] Hedstrom AK, Baarnhielm M, Olsson T, Alfredsson L (2009). Tobacco smoking, but not Swedish snuff use, increases the risk of multiple sclerosis.. Neurology.

[11] Hernan MA, Olek MJ, Ascherio A (2001). Cigarette smoking and incidence of multiple sclerosis.. Am J Epidemiol.

[12] Hernan MA, Jick SS, Logroscino G, Olek MJ, Ascherio A (2005). Cigarette smoking and the progression of multiple sclerosis.. Brain.

[13] Jafari N, Hoppenbrouwers IA, Hop WC, Breteler MM, Hintzen RQ (2009). Cigarette smoking and risk of MS in multiplex families.. Mult Scler.

[14] Riise T, Nortvedt MW, Ascherio A (2003). Smoking is a risk factor for multiple sclerosis.. Neurology.

[15] Silva KR, Alvarenga RM, Fernandez YFO, Alvarenga H, Thuler LC (2009). Potential risk factors for multiple sclerosis in Rio de Janeiro: a case-control study.. Arq Neuropsiquiatr.

[16] Pekmezovic T, Drulovic J, Milenkovic M, Jarebinski M, Stojsavljevic N (2006). Lifestyle factors and multiple sclerosis: A case-control study in Belgrade.. Neuroepidemiology.

[17] Rodriguez Regal A, del Campo Amigo M, Paz-Esquete J, Martinez Feijoo A, Cebrian E (2009). [A case-control study of the influence of the smoking behaviour in multiple sclerosis].. Neurologia.

[18] Russo C, Morabito F, Luise F, Piromalli A, Battaglia L (2008). Hyperhomocysteinemia is associated with cognitive impairment in multiple sclerosis.. J Neurol.

[19] Fowles J, Christophersen A, Fernando D, Lea R, Woodward A (2006). Secondhand tobacco smoke exposure in New Zealand bars: results prior to implementation of the bar smoking ban.. N Z Med J.

[20] Koch-Henriksen N, Sorensen PS (2010). The changing demographic pattern of multiple sclerosis epidemiology.. Lancet Neurol.

[21] Hawkes CH (2007). Smoking is a risk factor for multiple sclerosis: a metanalysis.. Mult Scler.

[22] De Jager PL, Chibnik LB, Cui J, Reischl J, Lehr S (2009). Integration of genetic risk factors into a clinical algorithm for multiple sclerosis susceptibility: a weighted genetic risk score.. Lancet Neurol.

[23] Healy BC, Ali EN, Guttmann CR, Chitnis T, Glanz BI (2009). Smoking and disease progression in multiple sclerosis.. Arch Neurol.

[24] Koch M, van Harten A, Uyttenboogaart M, De Keyser J (2007). Cigarette smoking and progression in multiple sclerosis.. Neurology.

[25] Sundstrom P, Nystrom L (2008). Smoking worsens the prognosis in multiple sclerosis.. Mult Scler.

[26] Pittas F, Ponsonby AL, van der Mei IA, Taylor BV, Blizzard L (2009). Smoking is associated with progressive disease course and increased progression in clinical disability in a prospective cohort of people with multiple sclerosis.. J Neurol.

[27] Clayton D, Hills M (1993). Statistical models in epidemiology: Oxford University Press..

[28] Bland JM, Altman DG (2000). Statistics notes. The odds ratio.. BMJ.

[29] Dahl J, Myhr KM, Daltveit AK, Skjaerven R, Gilhus NE (2007). Is smoking an extra hazard in pregnant MS women? Findings from a population-based registry in Norway.. Eur J Neurol.

[30] Frutos Alegria MT, Beltran-Blasco I, Quilez-Iborra C, Molto-Jorda J, Diaz-Marin C (2002). [The epidemiology of multiples sclerosis in Alcoi. Analytical data].. Rev Neurol.

[31] Frutos-Alegria MT, Beltran-Blasco I, Quilez-Iborra C, Molto-Jorda J, Diaz-Marin C (2002). [A control and case study of multiple sclerosis in the Alicante and Villajoyosa areas].. Rev Neurol.

[32] Senecal-Quevillon M, Duquette P, Richer CL (1986). Analysis of sister-chromatid exchanges (SCEs) in familial and sporadic multiple sclerosis.. Mutat Res.

[33] Turner AP, Kivlahan DR, Kazis LE, Haselkorn JK (2007). Smoking among veterans with multiple sclerosis: prevalence correlates, quit attempts, and unmet need for services.. Arch Phys Med Rehabil.

[34] Munger KL, Peeling RW, Hernan MA, Chasan-Taber L, Olek MJ (2003). Infection with Chlamydia pneumoniae and risk of multiple sclerosis.. Epidemiology.

[35] Munger KL, Chitnis T, Ascherio A (2009). Body size and risk of MS in two cohorts of US women.. Neurology.

[36] Nortvedt MW, Riise T, Maeland JG (2005). Multiple sclerosis and lifestyle factors: the Hordaland Health Study.. Neurol Sci.

[37] Villard-Mackintosh L, Vessey MP (1993). Oral contraceptives and reproductive factors in multiple sclerosis incidence.. Contraception.

[38] Hill AB (1965). The Environment and Disease: Association or Causation?. Proc R Soc Med.

[39] Sundstrom P, Nystrom L, Hallmans G (2008). Smoke exposure increases the risk for multiple sclerosis.. Eur J Neurol.

[40] Smith KJ, Kapoor R, Felts PA (1999). Demyelination: the role of reactive oxygen and nitrogen species.. Brain Pathol.

[41] Scolding N, Franklin R (1998). Axon loss in multiple sclerosis.. Lancet.

[42] George J, Levy Y, Shoenfeld Y (1997). Smoking and immunity: an additional player in the mosaic of autoimmunity.. Scand J Immunol.

[43] Green RM, Gally F, Keeney JG, Alper S, Gao B (2009). Impact of cigarette smoke exposure on innate immunity: a Caenorhabditis elegans model.. PLoS ONE.

[44] Fusby JS, Kassmeier MD, Palmer VL, Perry GA, Anderson DK (2010). Cigarette smoke-induced effects on bone marrow B-cell subsets and CD4+:CD8+ T-cell ratios are reversed by smoking cessation: influence of bone mass on immune cell response to and recovery from smoke exposure.. Inhal Toxicol.

[45] Motz GT, Eppert BL, Wortham BW, Amos-Kroohs RM, Flury JL (2010). Chronic cigarette smoke exposure primes NK cell activation in a mouse model of chronic obstructive pulmonary disease.. J Immunol.

[46] Mahdi H, Fisher BA, Kallberg H, Plant D, Malmstrom V (2009). Specific interaction between genotype, smoking and autoimmunity to citrullinated alpha-enolase in the etiology of rheumatoid arthritis.. Nat Genet.

[47] Graham NM (1990). The epidemiology of acute respiratory infections in children and adults: a global perspective.. Epidemiol Rev.

[48] Tremlett H, van der Mei IA, Pittas F, Blizzard L, Paley G (2008). Monthly ambient sunlight, infections and relapse rates in multiple sclerosis.. Neuroepidemiology.

[49] Lundstrom E, Kallberg H, Alfredsson L, Klareskog L, Padyukov L (2009). Gene-environment interaction between the DRB1 shared epitope and smoking in the risk of anti-citrullinated protein antibody-positive rheumatoid arthritis: all alleles are important.. Arthritis Rheum.

[50] Mack WP, Stasior GO, Cao HJ, Stasior OG, Smith TJ (1999). The effect of cigarette smoke constituents on the expression of HLA-DR in orbital fibroblasts derived from patients with Graves ophthalmopathy.. Ophthal Plast Reconstr Surg.

[51] Orton S-M, Herrera BM, Yee IM, Valdar W, Ramagopalan SV (2006). Sex ratio of multiple sclerosis in Canada: a longitudinal study.. Lancet Neurology.

[52] Ascherio A, Munger KL (2007). Environmental risk factors for multiple sclerosis. Part II: Noninfectious factors.. Ann Neurol.

