# Supplementary material for: Smoking and Multiple Sclerosis: An Updated Meta-Analysis
Source: PLoS One. 2011 Jan 13;6(1):e16149. doi: 10.1371/journal.pone.0016149 (PMC3020969; doi:10.1371/journal.pone.0016149)
Supplement: Table S1 — Characteristics of studies included in the meta-analysis. (DOC) [file pone.0016149.s001.doc]

| **First author & year of publication**  **(conservative/**  **non-conservative)** | **Type** | **Diagnostic criteria** | **Cases** | **Controls** | **Risk/odds ratio**  **(RR/OR)** | **Lower 95% CI** | **Upper 95% CI** | **Female/male ratio** | **Case source** | **Control source** | **Ascertainment** |
| --- | --- | --- | --- | --- | --- | --- | --- | --- | --- | --- | --- |
| **Susceptibility meta-analysis** | | | | | | | | | | | |
| Antonovsky 1965  (conservative) | Case-control | Not stated | 241 | 964 | 1.4 OR | 1.05 | 1.86 | 0.84 | Nationwide survey | Population controls | Questionnaire |
| Carlens 2010  (conservative) | Cohort | Medical record diagnosis | 214 | 277777 | 1.9 RR | 1.4 | 2.6 | All males | Prospective cohort study | Swedish construction cohort | Medical records |
| Da Silva 2009  (non-conservative) | Case-control | Specialist - including MRI & CSF | 81 | 81 | 2 OR | 0.9 | 4.3 | 2.12 | MS register | Neighbours & friends | Interview |
| Ghadirian 2001  (conservative) | Case-control | MS society association, neurologist & GP referrals | 197 | 202 | 1.6 OR | 1 | 2.4 | 2.17 | MS society, neurologist & GP referrals | Randomly chosen from population matched on age, sex and region of origin | Interview |
| Hedstrom 2009  (conservative) | Case-control | McDonald criteria | 902 | 1855 | 1.5 OR | 1.3 | 1.8 | 2.56 | Neurology clinic referrals | Randomly chosen from population matched on age, sex and region of origin | Questionnaire |
| Hernan 2001  (conservative) | Cohort | Poser criteria | 315 | 128638 | 1.6 RR | 1.2 | 2.1 | All females | Medical records, prospective cohort study | NHS I & II cohorts | Questionnaire |
| Hernan 2005  (conservative) | Case-control | Poser criteria | 201 | 1913 | 1.3 OR | 1 | 1.7 | 2.34 | Medical records, prospective cohort study | Randomly chosen from population matched on age, sex, GP practice & date of registration | Medical records |
| Jafari 2009  (conservative) | Case-control | McDonald criteria | 136 | 204 | 1.09 OR | 0.68 | 1.73 | 1.7 | MS database of familial MS | Unaffected siblings | Questionnaire |
| Pekmezovic 2006  (non-conservative) | Case-control | Poser criteria | 196 | 210 | 2.4 OR | 1.3 | 4.2 | 2.75 | Consecutive clinic referrals | Hernia patients | Questionnaire |
| Regal 2009  (non-conservative) | Case-control | McDonald criteria | 138 | 138 | 2.18 OR | 1.3 | 3.6 | 2.07 | Neurology clinic referrals | Emergency admissions | Unclear |
| Riise 2003  (conservative) | Case-control | Self-reported | 87 | 22312 | 1.81 RR | 1.13 | 2.92 | Not reported | Self-reported prevalence cases | Population controls | Questionnaire |
| Russo 2008  (non-conservative) | Case-control | Revised McDonald criteria | 94 | 53 | 1.11 OR | 0.51 | 2.42 | 1.24 | Consecutive clinic referrals | Healthy blood donors | Interview |
| Simon 2010  (conservative) | Case-control | Poser criteria & imaging | 136 | 272 | 1.5 OR | 1 | 2.4 | 2.13 | MS society & neurology referrals | Randomly selected from voter registration matched on age & sex | Questionnaire |
| Thorogood 1998  (conservative) | Cohort | Physician | 114 | 23000 | 1.2 RR | 0.8 | 1.8 | All females | Prospective cohort study | Prospective cohort | Interview |
| **Progression meta-analysis** | | | | | | | | | | | |
| Healy 2009 | Cohort | McDonald criteria | 1465 | N/A | 2.5 | 1.09 | 5.34 | 2.98 | MS centre | N/A | Follow-up visits |
| Hernan 2005 | Cohort | Poser criteria | 179 | N/A | 3.6 | 1.3 | 9.9 | 2.34 | GP database | N/A | Medical records |
| Koch 2007 | Cohort | Poser criteria | 364 | N/A | 0.89 | 0.6 | 1.32 | 2.11 | MS database | N/A | Follow-up visits |
| Sundstrom & Nystrom 2008 | Cohort | Not stated | 122 | N/A | 2.1 | 1.1 | 4 | 1.77 | Incident study | N/A | Medical records & interview |
